# Effects of low-level light therapy on dentin hypersensitivity: a systematic review and meta-analysis

**DOI:** 10.1007/s00784-021-04183-1

**Published:** 2021-10-13

**Authors:** Zhiyi Shan, Juanjuan Ji, Colman McGrath, Min Gu, Yanqi Yang

**Affiliations:** 1 Faculty of Dentistry, The University of Hong Kong, 34 Hospital Road, Sai Ying Pun, Hong Kong SAR, China; 2 Department of Stomatology, Second People’s Hospital of Yunnan Province, Kunming, People’s Republic of China

**Keywords:** Low-level light therapy, Dentin hypersensitivity, Systematic review, Meta-analysis

## Abstract

**Objective:**

To investigate the treatment efficacy of low-level light therapy on dentin hypersensitivity.

**Materials and methods:**

Following the PRISMA guideline, six electronic databases supplemented with bibliographies were searched till December 2020. Two reviewers performed the screenings independently with a reliability assessment. Studies fulfilling the pre-registered eligibility criteria were included for risk-of-bias assessment and data synthesis.

**Results:**

Thirty-five articles ultimately informed this systematic review based on the eligibility criteria and underwent *risk-of-bias* assessment (*ĸ* = 0.86). Quantitative results were deduced by meta-analysis of 20 randomised controlled trials: LLLT showed favourable outcomes compared to placebos for immediate (SMD: 1.09, 95% CI: 0.47 to 1.70), interim (SMD: 1.32, 95% CI: 0.41 to 2.23), and persistent efficacies (SMD: 2.86, 95% CI: 1.98 to 3.74). However, substantial heterogeneity existed among included studies (*I*^2^: 64–95%). Regarding comparisons with other desensitising strategies, LLLT showed no significant benefits in DH alleviation over others except fluorides for interim efficacy (SMD: 0.31, 95% CI: 0.10 to 0.52) and persistent efficacy (SMD: 0.45, 95% CI: 0.03 to 0.86).

**Conclusions:**

This systematic review shows that LLLT has positive immediate, interim, and persistent DH-treatment efficacies compared with placebo. No superior treatment effects of LLLT were observed except fluoride agent use. Further studies are warranted—RCTs with low risk of bias, consistent technical settings, comprehensive assessments, and long follow-up periods.

**Clinical relevance:**

This systematic review bridges a critical research gap by analysing clinical evidence in the DH-alleviating efficacy of LLLT in comparison with placebo and other in-office desensitising strategies.

**Supplementary Information:**

The online version contains supplementary material available at 10.1007/s00784-021-04183-1.

## Introduction

Dentin hypersensitivity (DH) is an unpleasant experience characterised by short and sharp dental pain in response to external stimuli that cannot be attributed to specific forms of dental defect or pathology [1, 2]. Twenty-five to 35% of the adult population have experienced DH [3, 4], and among those who suffer from periodontal diseases, the prevalence may be as high as 84% [5]. Although DH does not directly deteriorate tooth vitality or life expectancy, it is closely related to oral health–related functionality and may lead to physical, psychological, or social disability [6]. In recent decades, strategies to alleviate DH have been developed based on at-home management or professional clinical treatment [7]. However, none of these has met the criteria proposed by Grossman for an ideal DH treatment that addresses all aspects [8]: pulp integrity, rapid in action, permanent efficacy, comfortable and easy application, and no pigmentation on tooth structures [2, 9].

Home management with desensitising toothpaste is often the first-choice treatment for DH due to its wide availability and convenience for patients. However, the effects of this treatment usually take 4 to 8 weeks to develop [10]. Patients suffering from severe DH who desire immediate relief are highly recommended to seek professional care [11]. To date, a wide range of professional DH treatments has been introduced. The available modalities are typically classified in terms of their characteristics: varnishes and precipitants (e.g. fluorides, oxalates, calcium compounds, and bioactive glasses), restorative materials (e.g. adhesives, glass ionomers, and resins), agents for nerve desensitisation (such as potassium nitrates and guanethidine), light therapy, and periodontal surgery [9, 11, 12]. Despite this wide range of treatment choices, there is no consensus on which professional treatment is most effective or which treatment-application technique is most efficient [9].

Low-level light therapy (LLLT) refers to using red or near-infrared light to regulate biological activities without provoking thermal changes [13–16]. It is valued for its non-invasiveness, safety, comfort, precision, reproducibility, and rapid action [2, 17–19]. Chung et al. [14] suggested that the settings of LLLT are within 600–1070 nm wavelength and 1–1000 mW output power for good tissue penetration and promising treatment efficacy. Many clinical studies have reported the abilities of LLLT in DH alleviation. Yet, the effectiveness is still under debate: some studies corroborated findings that LLLT more effectively relieves DH than other strategies [20, 21], whereas others concluded that reductions in DH, especially those resulting in immediate relief, are substantially attributable to the placebo effect [22, 23]. A significant reason for the above inconsistency is the large variance in the technical parameters of light wavelength, beam size, output power, wave mode, exposure time, application frequency and irradiation method, and the periods of observation across studies [11, 19, 24]. The diversity of the comparators may also explain the inconsistent findings: some studies used negative controls, whereas others used positive controls since no gold-standard treatment has been established for DH management [11, 25, 26]. All above hinder the determination of the true efficacy of LLLT and its translation into clinical practice.

Therefore, this systematic review was conducted to analyse current evidence regarding the effects of LLLT on DH management. The primary outcome was treatment efficacies compared to placebo, based on the observed changes in patients’ subjective perceptions of DH at immediate (< 1 month), interim (1 to < 6 months), and persistent (≥ 6 months) time points. The secondary outcomes were the effects of LLLT on DH alleviation relative to those of other in-office desensitisation strategies, based on the evidence from previous clinical studies.

## Materials and methods

This systematic review was performed and is reported according to the Preferred Reporting Items for Systematic Reviews and Meta-Analyses (PRISMA) guidelines [27, 28]. The protocol was prospectively registered on the International Prospective Register of Systematic Reviews online database (CRD42020162721).

### Search strategy

Two reviewers (ZYS and JJJ) independently and systematically searched six major electronic databases (MEDLINE, EMBASE, PubMed, Scopus, ProQuest, and the Cochrane Central Register of Controlled Trials) from their date of establishment until December 2020 for manuscripts with English abstracts but no language restriction for the main text. The search terms used were medical subject headings, free text words, and their synonyms, and included ‘tooth/dentine/pulp’, ‘sensitivity/hypersensitivity/irradiation/discomfort/pain’. and ‘low-level light/low-intensity light/soft laser/cold laser/photobiomodulation’. Full details of this electronic searching strategy are presented in Appendix 1. Supplementary manual searching was performed by screening the bibliographies of all the included publications.

### Study selection

The eligibility criteria were as follows (in population, intervention, control, and outcomes format).

### Population

#### Inclusion criteria

1. Patients who self-reported DH.
2. Patients who had teeth with intact and vital pulps.
3. Systemically healthy patients with permanent dentition.

#### Exclusion criteria

1. Patients who had teeth containing cervical caries, defective restorations, premature contacts, cracked enamel, fluorotic damage, or any other factor that could be responsible for more exposed dentin tubules and DH.
2. Patients with teeth displayed any indication of pulpitis, pulp necrosis, or acute and chronic inflammation of the periapical and periodontal areas.
3. Patients who had teeth that had been subject to trauma, surgery, or invasive periodontal treatment within the past 3 months.
4. Patients who had DH while using desensitising toothpaste or receiving other dental treatments, such as dental bleaching, cavity or restorative preparation, or orthodontic treatment.
5. Patients who were pregnant or lactating were taking systemic medications or had severe craniofacial abnormalities, temporomandibular diseases, trigeminal neuralgia, or migraine that could affect their subjective judgement.

### Intervention

LLLT at a light wavelength between 600 and 1070 nm and an output power between 1 and 1000 mW [14].

### Comparison

Placebo or other in-office desensitisation strategies.

### Outcomes

Scores rated by patients for DH in response to external (thermal, chemical, tactile, electrical, or osmotic) stimuli.

### Study

Randomised controlled trials (RCTs) and non-randomised controlled studies (NRSs).

For literature management, all the titles and abstracts obtained from the electronic database searches were imported into *EndNote X9.3.3* software [29]. Two reviewers independently screened all the literature based on the eligibility criteria. Potentially relevant studies were retrieved for full-article assessment and final data synthesis. During the entire process, any disagreement between the two reviewers was resolved by discussion or consultation with a third reviewer (YQY). Cohen’s *κ*-values were computed to verify inter-reviewer reliability, and *κ* 0.6 was considered to indicate acceptable reliability [30].

### Data extraction and analysis

The following data were extracted: general information (first author, nationality, and year of publication), study type and design, participants (number, age, and sex) and target teeth, intervention (light’s type, wavelength, wave mode, output power, energy density, time of exposure, irradiation session, total dosage, and method of irradiation), comparators, and outcome assessment (stimulus, numeric scale, and observation period).

### Risk-of-bias assessment

The risk-of-bias assessment was performed in *RevMan.5.4* [31], according to the Cochrane Handbook [32]. RCTs were evaluated using the revised Cochrane risk-of-bias tool for randomised trials (RoB 2) [33] in the following five domains: bias from the randomisation process, bias due to deviations from the intended intervention, bias due to missing outcome data, bias in the measurement of the outcome, and bias in the selection of the reported result. NRSs were assessed using the Risk of Bias in Non-randomised Studies of Interventions (ROBINS-I) tool [34] in the following seven domains: bias due to confounding, bias in the selection of participants for the study, bias in the classification of interventions, bias due to deviations from the intended intervention, bias due to missing data, bias in the measurement of outcomes, and bias in the selection of the reported result. Following the assessment of all domains, each study’s overall risk of bias was graded according to the Handbook as ‘low, some concerns, or high’ (for RCTs) and ‘low, moderate, serious, or critical’ (for NRSs). The two reviewers (ZYS and JJJ) conducted this process independently, and any disagreements were resolved by discussion.

### Data synthesis and statistical analysis

Quantitative syntheses of data from RCTs and NRSs with a low risk of bias were performed according to the guidelines in the Cochrane Handbook [35]. Based on the results of data extraction, the effects of LLLT on the changes in DH, as indicated by patients’ self-rated scores on a visual analogue scale (VAS; 0 to 100) immediately after LLLT sessions (first assessment post-treatment), at interim follow-ups (last assessment within 1 month and up to 6 months) and persistent follow-ups (last observation at 6 months or beyond), were collected and pooled. The results of studies that used other numeric scales were transformed proportionally to VAS scores using a standard formula: $VAS score=\frac{{x}_{i}}{\mathrm{max}\left({x}_{i}\right)}\times 100$, where ${x}_{i}$ were readings of $i$-th numeric scale and $\mathrm{max}(\cdot )$ denoted the maximum element of the scale. Ultimately, this yielded all data on one generic VAS (0 to 100; 0 = no pain, 100 = worst possible pain) for meta-analysis. Since there are considerable clinical-setting variations in participants’ age and gender, LLLT’s technical parameters, and DH assessment approaches, the outcomes were analysed using *RevMan5.4* [31] by pooling standard mean differences (SMDs) and 95% confidence intervals (CIs) of individual studies based on a random-effects model to minimise the impact of precision variance among studies [36]. The results are presented in forest plots and a summary-of-findings table. Statistical heterogeneity was evaluated using the *I*^2^ statistic, and *I*^2^ values > 50% were considered to indicate substantial or critical heterogeneity. Based on the sufficiency of pooled data, a multiple meta-regression was conducted using *Stata 15 software* [37] to analyse the efficacy of LLLT on DH alleviation, with adjustment for factors associated with study quality and interventional settings.

## Results

**Fig. 1:**
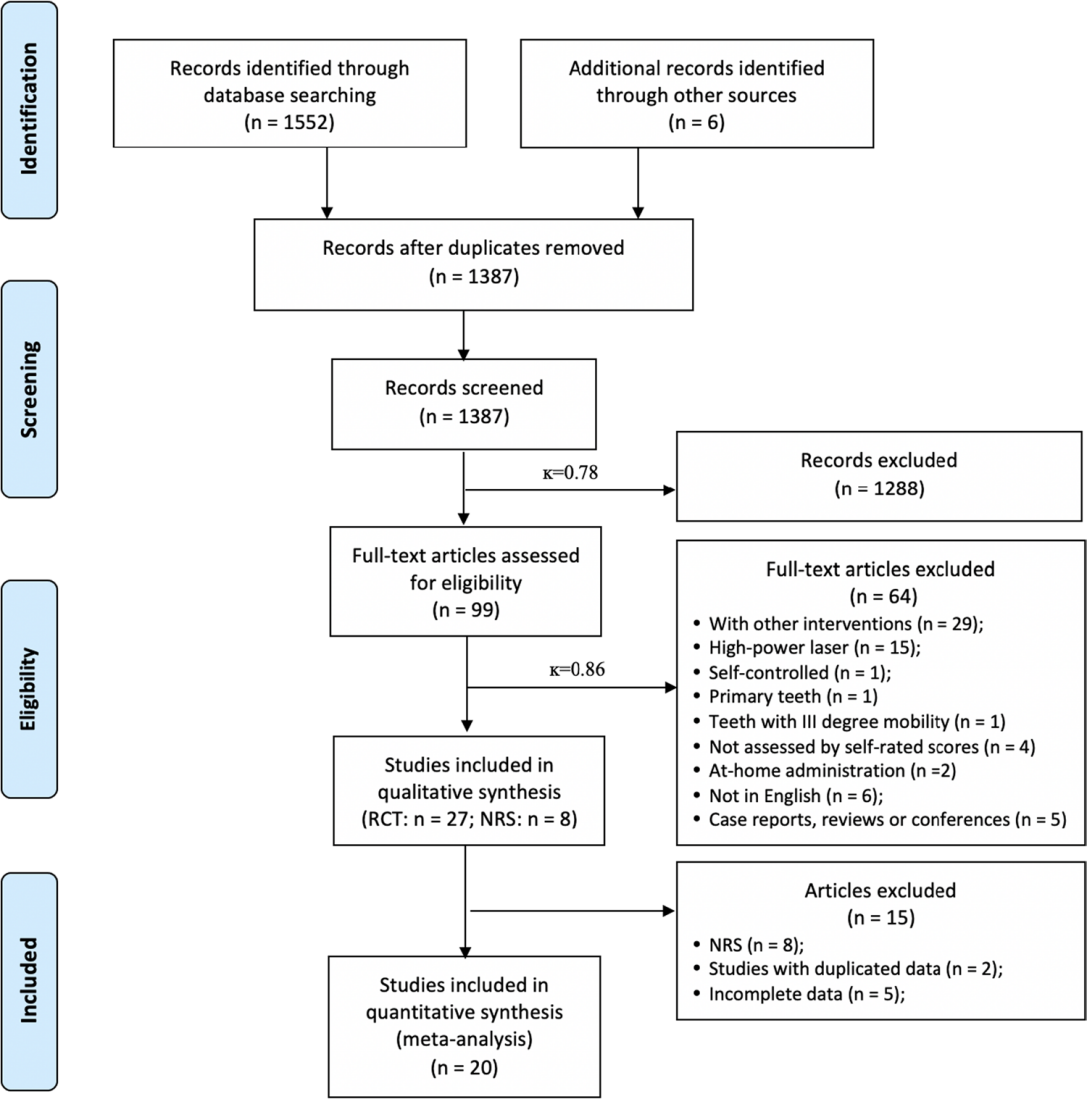
PRISMA flow diagram

The electronic searches of the six databases, supplemented with manual searching, yielded 1558 records. Following the removal of duplicates, the titles and abstracts of 1387 records were screened according to our pre-registered eligibility criteria. This yielded 99 articles for full-text assessment (*ĸ* = 0.78). Following assessment of these articles according to the eligibility criteria, 64 studies were excluded, and 35 articles were included in the qualitative data synthesis (*ĸ* = 0.86), comprising 27 RCTs [20–23, 38–60] and eight NRSs [17, 61–67]. Subsequent quantitative data syntheses were performed using data from 20 RCTs that reported the same outcome for DH alleviation, as measured by numeric scales according to patients’ self-perceptions in response to chair-side air blast stimuli. All eight NRSs [17, 61–67] were excluded due to a moderate-to-serious risk of bias. In addition, two RCTs that contained duplicated data [49, 59] and five RCTs that had incomplete data [38, 42, 50, 52, 53] were excluded. The entire study-selection procedure is illustrated in the PRISMA flow diagram depicted in Fig. 1.

### Characteristics of included studies

All the study samples comprised subjects of both sexes over a wide age range (12–70 years). The interventions consisted of a diode laser [17, 21, 22, 38, 39, 42–47, 49–52, 54–65, 67] or a neodymium-doped yttrium aluminum garnet laser (Nd:YAG) [21, 23, 40, 41, 43, 44, 51, 53, 65, 66] and were delivered using a wide range of parameters in terms of wavelength (630–1067 nm), output power (1.5–1000 mW), total dosage (0.1–300 J), energy density (2–100 J/cm^2^), exposure time (10–180 s) and number of irradiation sessions (1–6). Overall, 12 studies compared the effects of LLLT with placebo [22, 23, 39, 40, 44, 47, 52, 53, 55, 57, 59, 60], and the other comparators were fluorides [21, 38, 42, 45, 52, 58, 60, 61, 64], adhesives [20, 38, 45, 46, 49–51, 55, 56], potassium nitrate or oxalate [22, 38, 54, 56, 57], and dentifrices (arginine-calcium carbonate [39] and calcium sodium phosphosilicate [23]). Most studies examined the outcome of DH treatment by the patient’s subjective response to an air blast as determined by VAS or other numeric scales, namely a 3-point [49], 4-point [41, 53, 57, 65, 67], or 5-point scale [64]. The other DH investigations included response to mechanical [22, 23, 40–42, 47–51, 53, 54, 57, 58, 62], ice-cold [20, 46, 54, 63, 66], and electric [54] stimuli. The detailed characteristics of all included RCTs and NRSs are illustrated in Table 1 and Table 2, respectively.

**Table 1:** Characteristics of included RCTs (*RCTs*, randomised controlled trial; *SMD*, split-mouth design; *M*, males; *F*, females; *PW*, pulse wave; *CW*, continuous wave; *VAS*, visual analogue scale; *y*, years; *m*, months; *d*, days; *G*, group; *LLLT*, low-level light therapy)

Study ID	Design	Participants/target teeth	LLLT type	Wavelength (nm)	Mode	Output power (mW)	Energy density per irradiation(J/cm^2^)	Time of exposure (s)	Irradiation session (times)	Total dosage (J)	Methods for irradiation	Assessment	Comparisons	Outcomes
Aranha 2009 (Brazil) [38]	RCT; parallel	39p; 101 teeth;	GaAlAs	660	CW	15	3.8	40	3	1.8	Perpendicularly to the tooth surface at four points in contact mode (3 cervical and 1 apical)	VAS (air)	LLLT; Gluma; Seal&Protect; Potassium oxalate; Fluoride;	**↓** (*p* < 0.05) for all groups;
Bal 2015 (Turkey) [39]	RCT; SMD	21p (5 M,16F; 19–60y); 156 teeth;	Diode	685	PW (9 Hz)	25	2	80	1	2	Fiber tip at a distance of 2 mm from the dental outer surface	VAS (air)	LLLT; Arginine-calcium carbonate LLLT + ACC; ACC + LLLT; placebo;	**↓** (*p* < 0.05) for G1 to G4; 90d: G1 (72%), G2 (65.4%), G3 (54.6%), G4 (69.6%), G5 (− 7.8%);
Birang 2007 (Iran) [40]	RCT, SMD	9p (5 M,4F); 63 teeth	Nd:YAG	1064	PW (15 Hz)	1000	NA	60	2	120	Not specified	VAS (air and probe)	Nd:YAG; Er:YAG; Placebo;	**↓** (*p* < 0.05) for all groups;
Bou Chebel 2018 (Lebanon) [41]	RCT, SMD	12p (20–60y); 54 teeth;	Nd:YAG	1064	PW (2 Hz)	640	35.8	20	4	51.2	With scanning movements in mesiodistal directions 6 mm distance away from dentinal surfaces	4-point scale (air); VAS; tactile score; thermal test;	LLLT; Sodium fluoride	**↓** (*p* < 0.05) for all groups; 1w: 79% (G1), 69.6% (G2); 6 m: 61% (G1), 46% (G2);
Dantas 2016 (Brazil) [42]	RCT, parallel	86 teeth	GaAlAs	808	CW	NA	4	NA	4	NA	Punctually to the cervical region on the buccal face	VAS (air and probe)	LLLT; Fluoride;	**↓** (*p* < 0.05) 6 m: to air 81% (G1) and 67.1% (G2); to probe 83.1% (G1) and 63.4% (G2)
Dilsiz 2009 (Turkey) [43]	RCT, SMD	14p (6 M,8F; 19–51y); 56 teeth;	Diode; Nd:YAG	685; 1064;	PW (10 Hz)	25 1000	2 NA	100 60	3	7.5 180	Diode laser: in a continuous mode on the buccal neck with exposed dentine; Nd:YAG: in a sweeping mode and 2 mm above tooth surface	VAS (air)	Diode laser; Nd:YAG	Session 1: G1:↓ (*p* > 0.05), G2:**↓** (*p* < 0.01) Session 2: **↓** (*p* < 0.05) for both groups Session 3: **↓** (*p* < 0.05) for both groups Follow-up: **↓** (*p* < 0.05) for both groups
Dilsiz 2010 (Turkey) [44]	RCT, SMD	24p (11 M,13F;18–52y); 96 teeth;	GaAlAs; Nd:YAG	808; 1064;	CW; PW (15 Hz)	100 1000	NA	40 100	3	12 300	2 mm from the surface in scanning movements perpendicularly to the region of the exposed dentinal neck	VAS (air)	GaAlAs; Er:YAG; Nd:YAG; Control;	Session 1–3: ↓ (*p* < 0.05) for all treatment groups except the third application of GaAlAs (*p* > 0.05); Follow-up on days 15, 30, and 60: ↓ (*p* < 0.05) maintained for all treatments but not change over time
Femiano 2013 (Italy) [45]	RCT, SMD	24p (8 M,16F; 21–64y); 262 teeth;	Diode	808	CW	200	NA	60	3	36	At a distance 0.5–1.0 cm in rapid movements to tooth surfaces perpendicularly	VAS (air)	LLLT; NaF; NaF + LLLT; Gluma;	**↓** (*p* < 0.05) except for NaF at 6 m; Immediately: (G1–G4) 72.2%, 51.6%, 82.6%, 77.4%; 1 m: 62.5%, 29.7%, 69.5%, 56.1%; 6 m: 47.2%, 4.7%, 60.8%, 27.3%;
Flecha 2013 (Brazil) [46]	RCT, SMD	62p (15 M,47F; 12–60y); 434 teeth;	GaAlAs	795	CW	120	2.88	24	3	8.64	At three points around the neck of the tooth	VAS (air and cold spray)	LLLT; Cyanoacrylate	**↓** (*p* < 0.05)
Gentile 2004 (Brazil) [47]	RCT, parallel	32p (10 M,22F; 20–52y); 68 teeth	GaAlAs	670	CW	15	4	120	6	10.8	Punctual application of the laser at three points (distal, central, and mesial), and with the intraoral tip positioned perpendicular to the dentin surface	VAS (air and probe)	LLLT; Placebo	**↓** (*p* < 0.05)
Gerschman 1994 (Australia) [48]	RCT, parallel	71p (15–69y); 71 teeth	GaAlAs	830	CW	30	NA	60	3	5.4	Not specified	VAS (air and probe)	LLLT; Placebo;	VAS_G1 < VAS_G2 (*p* = 0.002 for tactile stimulus; *p* < 0.001 for air blasts)
Lima 2017 (Brazil) [49]	RCT, SMD	62p (15 M,47F; 12–60y) 432 teeth;	GaAlAs	795	CW	120	30.96	24	3	8.64	In contact mode at three points around the cervical region of the tooth	OHIP14; 3-point scale (air and probe)	LLLT; Cyanoacrylate	**↓** (*p* < 0.05) in OHIP14; 180d: 80.6% of participants reported an improvement in their conditions;
Lopes 2015 (Brazil) [50]	RCT; parallel	27p (22–53y); 55 lesions	GaAlAs	810	CW	30; 100;	10; 90;	36; 22;	3	2.4; 15;	Perpendicular to the surface and in contact with the tooth cervical or apical surfaces	VAS (air and probe)	Gluma densensitizer; LLLT with low dose; LLLT with high dose; LLLT (low) + Gluma; LLLT (high) + Gluma;	**↓** (*p* < 0.05) for all groups; Air (immediately after and 6 m): G1 (59%, 65%), G2 (60%, 71%), G3 (87%, 87%), G4 (79%, 81%), G5 (75%, 83%); Probe (immediately after and 6 m): G1 (89%, 90%), G2 (78%, 86%), G3 (93%, 81%), G4 (93%, 90%), G5 (82%, 96%);
Lopes 2017 (Brazil) [51]	RCT, parallel	32p (22–53y); 117 teeth;	GaAlAs Nd:YAG	810; 1064;	CW PW (10 Hz)	30, 100; 1000;	10; 40; 85;	36; 22; 60;	3; 3; 1;	3.24; 6.6; 60;	In contact mode perpendicular to the tooth surface	VAS (air and probe)	Gluma; Diode (low); Diode (high); Diode (low) + Gluma; Diode (high) + Gluma; Nd:YAG Nd:YAG + Gluma; Diode (low) + Nd:YAG; Diode (high) + Nd:YAG;	**↓** (*p* < 0.05) for all groups;
Lund 2013 (Brazil) [52]	RCT, parallel	13p (5 M, 8F; 19–58y); 117 teeth	GaAlAs	780	CW	20	5	40	3	2.4	Four punctual applications, three at the cervical zone and one at the root apex	VAS (air) Exposure time (air)	LLLT; Fluoride; Placebo;	**↓** (*p* < 0.05) for all groups;
Maximiano 2019 (Brazil) [23]	RCT, parallel	70p (18–65y); 394 teeth;	Nd:YAG	1064	PW (10 Hz)	1000	85	15	4	60	Four irradiations were made with scanning movements: two in the mesiodistal and two in the occlusal-gingival directions	VAS (air and probe)	Nd:YAG; Calcium Sodium Phosphosilicate; Placebo;	↓ (*p* < 0.05) for all groups;
Mogharehabed 2012 (Iran) [53]	RCT, SMD	9p (3 M, 6F); 60 teeth;	Nd:YAG	1064	PW (20 Hz)	1000	NA	120	1	120	At a distance of 3 mm without cooler	4-point scale (air); VAS (probe); EPT;	Placebo; NaF; LLLT; NaF + LLLT;	↓ (*p* < 0.05) for all treatment (except G1);
Narayanan 2019 (Saudi Arabia) [54]	RCT, parallel	45p (68 M,22F; 18–60y); 264 teeth;	Diode	810	CW	1000	NA	10	1	10	With the appliance tip placed tangentially to the tooth surface and 1 mm away from it	VAS (air, cold water, electrical tactile);	Potassium nitrate; LLLT; LLLT + potassium; nitrate;	**↓** (*p* < 0.05) in G3 3 m: to air G1: 1.3%, G2: 24.4%, G3: 51.5%; to ice water G1: 6.4%, G2: 36.5%, G3: 46.9% to electrical tactile G1: − 11.3%, G2: 39.8%, G3: 54.4%
Orhan 2011 (Turkey) [55]	RCT, parallel	16p (8 M,8F; 21–51y); 64 teeth	GaAlAs	655	CW	25	4	160	6	24	in continuous mode with contact on the region of exposed dentinal area in a uniform, sweeping, and scanning motion	VAS (air)	Gluma; LLLT; Distilled water; Placebo LLLT;	↓ (*p* < 0.05) for G1 and G2; 24 h: G1: 40%; G2: 44%; G3: 3.7%; 0%; 7d: G1: 85%; G2: 87%; G3: 3.7%; 0%;
Osmari 2018 (Brazilz) [56]	RCT, SMD	19p (6 M,13F; 21–48y); 76 teeth;	Diode	810–830	CW	1000	100	20	1	20	At a distance of 1 mm from the dentinal surface with horizontal scanning movements	VAS (air)	NaF; Potassium oxalate; Adhesive; LLLT;	**↓** (*p* < 0.05) for G4 from 15 days onward
Praveen 2019 (India) [20]	RCT, parallel	23p; 50 teeth;	LLLT	904	PW (4000 Hz)	60	9	180	1	7.2	Perpendicular to tooth surface at three points, and as close as possible with the tooth surface without contact	VAS (air and cold water)	LLLT; Gluma;	↓ (*p* < 0.05) for all treatment
Sicilia 2009 (Spain) [57]	RCT, parallel	45p (18 M,27F; 19–70y);	GaAlAs	810	CW	1.5–2.5	NA	60	1	0.09–0.15	Not specified	4-point scale (air and tactile); 6-point scale (daily life);	LLLT; Potassium nitrate; Placebo;	↓ (*p* < 0.05) for all treatment; 30 min: G1: 39.9%, G2: 5.4%, G3: 12.7%; (*p* = 0.004) 14 days: G1: 71.7%, G2: 36.3%, G3: 28.1%; (*p* = 0.004) 60d: G1: 65.7%, G2: 30.4%, G3: 25.8%; (*p* = 0.01)
Soares 2016 (Brazil) [21]	RCT, parallel	23p (3 M,20F; 20–65y); 89 teeth;	GaAlAs Nd:YAG	810; 1064	CW; PW (10 Hz)	40; 1000	4; NA	60	1	60; 2.4;	Nd:YAG: to the cervical surface in non-contact mode; GaAlAs: in a contact mode on four points	VAS (air)	Nd:YAG; GaAlAs; 2% fluoride gel;	↓ (*p* < 0.05) for all treatment at all the time intervals; Immediate: G1 (93.75%), G2 (100%), G3 (81.2%); 1w: G1 and G2 (100%), G3 (81.25%);
Umberto 2012 (Italy) [58]	RCT, SMD	10p (2 M, 8F; 25–60y); 115 teeth;	GaAlAs	980	CW	500	62.2	60	3	90	In non-contact mode	VAS (air and probe)	NaF; LLLT; NaF + LLLT;	↓ (*p* < 0.05) for all treatment; To air: 10.19% (G1); 22.35% (G2); 25.04% (G3); To tactile: 4.13% (G1); 6.77% (G2); 9.96% (G3);
Vieira 2009 (Brazil) [22]	RCT, parallel	30p (7 M,23F; 24–68y); 164 teeth;	GaAlAs	660	CW	30	4	120	4	14.4	Perpendicularly to the tooth surface at four points (3 cervical and 1 apical)	VAS (air and probe)	LLLT; Potassium oxalate; Placebo;	↓ (*p* < 0.05) for all treatment at all the time intervals
Yilmaz 2011–1 (Turkey) [59]	RCT, SMD	51p (22 M,29F; 44 ± 9.7y); 174 teeth	GaAlAs	810	CW	500	8.5	60	1	30	Scanning the cervical part in an overlapping pattern	VAS (air)	Er,Cr:YSGG; GaAlAs; Placebo;	↓ (*p* < 0.05) for G1 and G2
Yilmaz 2011–2 (Turkey) [60]	RCT, SMD	48p (22 M,26F; 18–58y); 244 teeth	GaAlAs	810	CW	500	8.5	60	1	30	Scanning the cervical part in an overlapping pattern	VAS (air)	LLLT; NaF; Placebo LLLT; Placebo NaF;	↓ (*p* < 0.05) for G1 and G2

**Table 2:** Characteristics of included NRSs (*NRSs*, controlled-clinical trials; *SMD*, split-mouth design; *M*, males; *F*, females; *PW*, pulse wave; *CW*, continuous wave; *VAS*, visual analogue scale; *y*, years; *m*, months; *d*, days; *G*, group; *LLLT*, low-level light therapy)

Study ID	Design	Participants/target teeth	LLLT type	Wavelength (nm)	Mode	Output power (mW)	Energy density per irradiation (J/cm^2^)	Time of exposure (s)	Irradiation session (times)	Total dosage (J)	Methods for irradiation	Assessment	Comparisons	Outcomes
Corona 2003 (Brazil) [61]	NRS; SMD	12p (20–30y); 60 teeth;	GaAlAs	660	CW	15	4	30	5	2.25	Perpendicularly to tooth surface at three points (one apical and two cervical)	VAS (air)	LLLT with 3 J/cm^2^; LLLT with 5 J/cm^2^;	**↓** (*p* < 0.05) 60d: 86.53% (G1) and 88.88% (G2)
Hashim2014 (Sudan) [62]	NRS, parallel	5p (2 M, 3F; 25–35y); 14 teeth;	GaAlAs	810	CW	1000	NA	30; 60;	2	60; 120;	In non-contact mode at the cervical region	VAS (probe)	LLLT with 30 s LLLT with 60 s	**↓** (*p* < 0.05) for both G1 and G2; 15 min: VAS_G2 < VAS_G1 (*p* = 0.002); 7d: both dropped to 0;
Ladalardo 2004 (Brazil) [63]	NRS, parallel	20p (9 M,11F; 25–45y); 40 teeth	GaAlAs	660; 830	CW	35	4	114	4	16	Punctually applied with contact mode on the region of exposed dentinal buccal neck	VAS (cold nociceptive stimulus of 0℃)	LLLT in 660 nm; LLLT in 830 nm;	**↓** (*p* < 0.05)
Marsilio 2003 (Brazil) [17]	NRS, parallel	25p (14–58y); 106 teeth	GaAlAs	670	CW	15	3; 5;	114; 190;	6	10.26; 17.1;	At buccal cervical, approximately 3 mm away in a perpendicular direction to the cemento-enamel junction	VAS (air)	LLLT with 3 J/cm^2^; LLLT with 5 J/cm^2^;	**↓** (*p* < 0.05) for all groups; 60d: 86.53% G1 and 88.88% G2
Pesevska 2010 (USA) [64]	NRS, parallel	30p (25–40y);	Diode	630–670	CW	15	6	40	3	1.8	Directed perpendicularly to tooth surface at two points	4-point scale (daily life)	LLLT; Fluoride;	Session1: G1 27% and G2 0%; Session2: G1 87% and G2 27%;
Tabatabaei 2018 (Iran) [65]	NRS, parallel	22p (25–58y); 135 teeth;	GaAlAs Nd:YAG	810; 1064;	CW; PW (10 Hz)	200; 1000;	89.4; 49,760;	30; 40;	3	18; 120;	With the sweeping motion of the tip of laser hand piece to the cervical area	4-point scale (air)	GaAlAs; Nd:YAG; Bonding agent;	↓ (*p* < 0.05) for all treatment at immediately after and 1 month; ↓ (*p* < 0.05) for G2 at 3 m and 6 m post-tx;
Talesara2014 (India) [66]	NRS, SMD	20p (10 M,10F; 25–55y); 80 teeth;	Nd:YAG	1064	PW (10 Hz)	1000;	NA	60	2	120	With 2 mm away from the tooth surface	VAS (air and cold water)	Potassium binoxalate gel; LLLT;	↓ (*p* < 0.05) for all treatment at all the time intervals
Tengrungsun 2008 (Thailand) [67]	NRS, SMD	70p (20–60y); 140 teeth;	GaAlAs	790	CW	30	NA	60	1	1.8	Not specified	4-point scale (air)	GaAlAs; Bond agent;	↓ (*p* < 0.05) for all treatment at all the time intervals

### Risks of bias

**Fig. 2:**
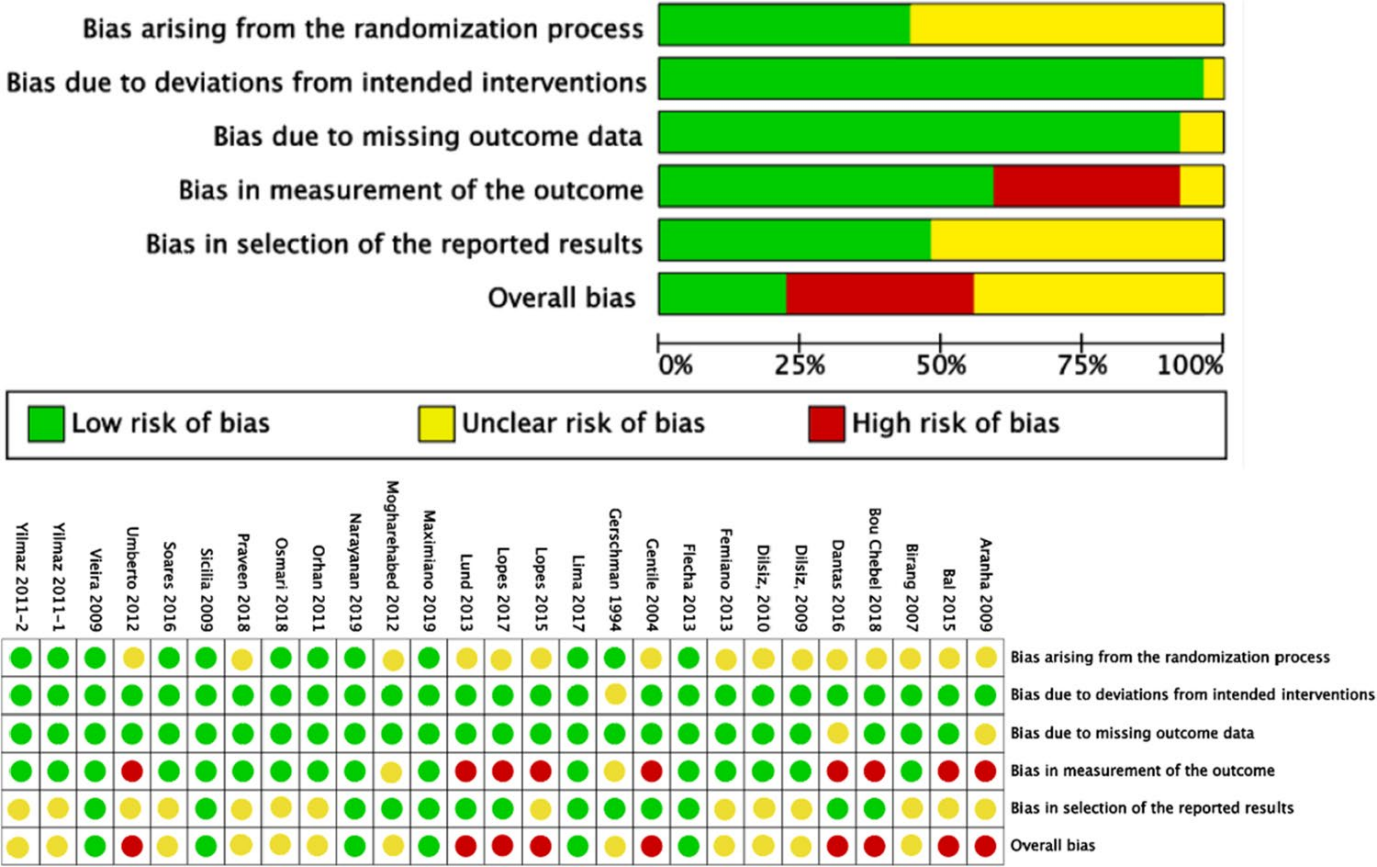
Risk-of-bias assessment of twenty-seven randomised controlled trials (RCTs) with A Revised Cochrane Risk-of-Bias Tool for Randomized Trials (RoB 2)

The risks of bias in the 27 RCTs was evaluated in five domains using the RoB 2 Tool [33]. As shown in Fig. 2, nine studies had a high risk of bias that was mainly arising from outcome measurements [38, 39, 41, 42, 47, 50–52, 58]. A further 12 studies were rated as having ‘some concerns’ in the overall risk of bias, as they possessed an unclear risk of bias in at least one domain arising from randomisation or selection of reported results [20, 21, 40, 43–45, 48, 53, 55, 56, 59, 60], and six studies presented a low risk of bias across all domains [22, 23, 46, 49, 54, 57].

**Fig. 3:**
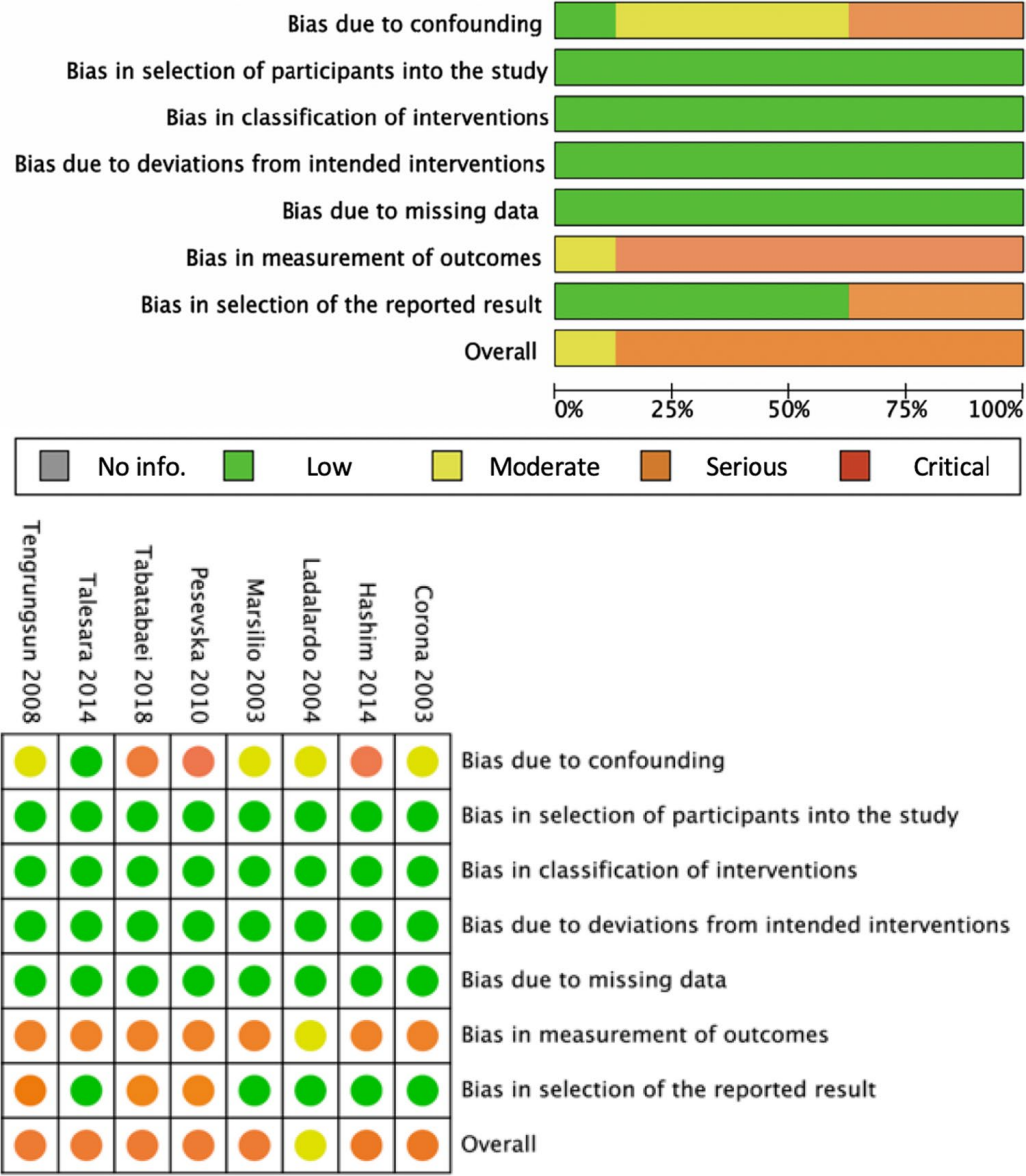
Risk-of-bias assessment of eight non-randomised controlled trials (NRSs) with Risk of Bias Tool in Non-Randomized Studies – of Interventions (ROBINS-I)

The overall risks of bias in the eight NRSs were assessed in seven domains with four levels (low, moderate, serious and critical) using the ROBINS-I Tool [34]. As shown in Fig. 3, one NRS had a moderate risk of bias [63] and the remaining seven studies had a serious risk of bias [17, 61, 62, 64–67]. All eight NRSs were excluded from the subsequent meta-analysis.

### Meta-analysis

When processing meta-analysis, we noticed that the included studies were various in stimuli devices and application methods, making it challenging to synthesise studies using other external stimuli than air blasts quantitatively. Therefore, only studies using air blast stimuli were included in quantitative meta-analysis. Quantitative analysis of LLLT’s effect on DH was based on the changes in VAS score (0 to 100; 0 = no pain, 100 = worst possible pain). Eighteen studies [20–23, 39, 40, 43–48, 51, 54–56, 58, 59] used VAS, and only two studies [41, 57] evaluated DH by a 4-degree scale and needed proportional transformation to a VAS. For these two studies, a standard formula was used. Since this conversion may bring up some precision variances in the data extracted, we presented the results in SMDs based on the random-effects model, which is a classical way to minimise the influence of precision variances [36].

#### DH-alleviating efficacy of LLLT compared to placebo

**Fig. 4:**
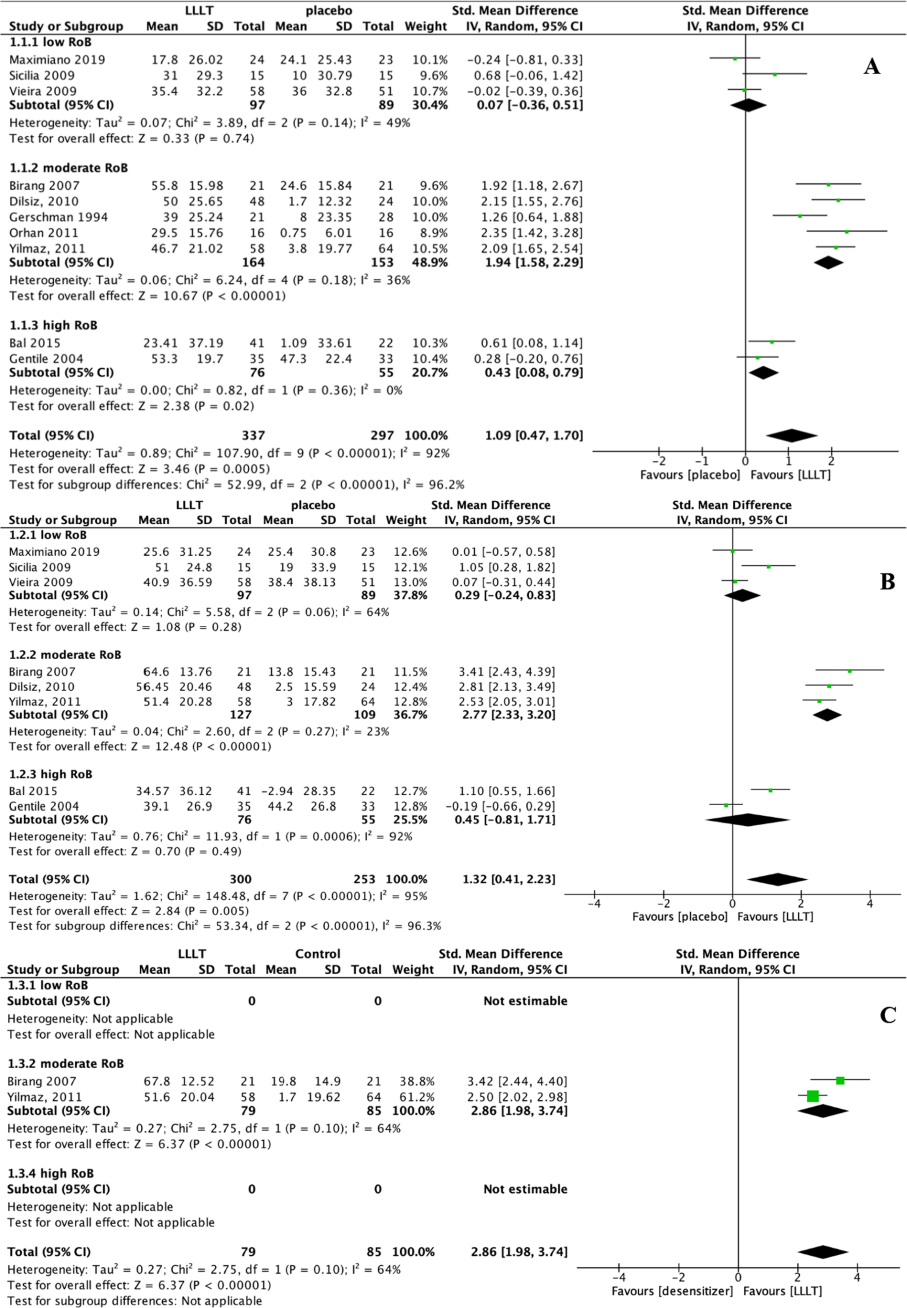
Forest plots indicating treatment efficacy of LLLT on DH alleviation compared to placebo effect: **A** immediate efficacy; **B** interim efficacy; **C** persistent efficacy

The results show that compared to placebo, LLLT alleviated DH at all stages. In terms of immediate efficacy, the SMD between LLLT and placebo was 1.09 (95% CI: 0.47 to 1.70, *p* < 0.001). In terms of interim efficacy and persistent efficacy, the SMD between LLLT and placebo was 1.32 (95% CI: 0.41 to 2.23, *p* = 0.005) and 2.86 (95% CI: 1.98 to 3.74, *p* < 0.001), respectively (Table 3). Interestingly, there was a significant difference between the immediate and interim efficacy SMDs in a subgroup analysis of studies categorised by the risk-of-bias level (i.e. low, moderate, or high) (*p* < 0.001). No study with persistent efficacy had a low risk of bias. The statistical heterogeneity was assessed by determining the *I*^2^ values for all included studies in terms of immediate, interim, and persistent efficacies, which were 92%, 95%, and 64%, respectively (Fig. 4). Funnel plots show that publication bias existed for all periods. Due to the high *I*^2^ and considerable variability in the technical parameters used in different studies regarding wavelength, output power, wave mode, exposure time, application frequency, and irradiation method, a meta-regression was conducted to determine the true ability of LLLT to alleviate DH and the related factors (covariates).

**Table 3:** Summary-of-findings table for the effects of LLLT on DH alleviation in comparison with placebo effect

LLLT for dentin hypersensitivity						
**Patient or population:** patients with dentin hypersensitivity **Settings:** in-office administration **Intervention:** LLLT **Comparison:** placebo						
**Outcomes**	Anticipated absolute effects* (95% CI)		**SMD (95% CI)**	**No. of participants (studies)**	**Quality of the evidence (GRADE)**	**Comments**
	**Placebo**	**LLLT**				
**Immediate efficacy **0 day to less than 1 month* (VAS scale 0 to 100, 0* = *no pain, 100* = *worst possible pain)*	The mean immediate reduction of VAS score ranged across placebo groups from **0.75 to 47.3**	The mean immediate efficacy ranged across LLLT groups was **22.94 points lower** (34.44 to 11.43 lower)	1.09 (0.47–1.70)	634 (10 studies)	⨁⨁◯◯ LOW	Inconsistency↓ Imprecision ↓ Publication bias↓ Large magnitude of effect ↑
**Interim efficacy** 1 month to less than 6 months* (VAS scale 0 to 100, 0* = *no pain, 100* = *worst possible pain)*	The mean interim reduction of VAS score ranged across placebo groups from − **2.94 to 44.2**	The mean interim efficacy ranged across LLLT groups was **38.44 points lower** (42.25 to 34.62 lower)	1.32 (0.41–2.23)	553 (8 studies)	⨁⨁◯ LOW	Inconsistency↓ Imprecision ↓ Publication bias↓ Large magnitude of effect↑
**Persistent efficacy **6 months and after* (VAS scale 0 to 100, 0* = *no pain, 100* = *worst possible pain)*	The mean persistent reduction of VAS score ranged across placebo groups was **1.7 to 19.8**	The mean persistent efficacy ranged across LLLT groups was **49.11 points lower** (54.49 to 43.73 lower)	2.86 (1.98–3.74)	164 (2 studies)	⨁◯◯◯ VERY LOW	Limitation in study design or execution↓ Inconsistency↓ Imprecision↓ Publication bias↓ Large magnitude of effect↑
*GRADE* working group grades of evidence **High quality:** further research is very unlikely to change our confidence in the estimate of effect **Moderate quality:** further research is likely to have an important impact on our confidence in the estimate of effect and may change the estimate **Low quality:** further research is very likely to have an important impact on our confidence in the estimate of effect and is likely to change the estimate **Very low quality:** we are very uncertain about the estimate

The meta-regression of immediate and interim efficacies was performed using *Stata 15 Software* [37]*,* and five factors were assessed: ‘risk of bias’, ‘wavelength’, ‘wave mode’, ‘energy density’, and ‘total dosage’. Due to data insufficiency for long-term follow-ups, a meta-regression of persistent efficacy could not be conducted. In addition, three factors—‘output power’, ‘time of exposure’, and ‘irradiation sessions’—were not individually investigated, as they have multiplicative relationships with the ‘total dosage’, according to the following equation:

$$Total dosage=Output power\times Time of exposure\times Irradiation Sessions$$

The results of a random-effect model analysis using a forward method reveal that only ‘energy density’ is significantly correlated with the immediate and interim treatment effects of LLLT, as demonstrated by the adjusted *R*^2^ values of 34.71% and 60.11%, respectively. The residual variances (*I*^2^_*res*_) due to heterogeneity are 83.89% for immediate efficacy and 49.11% for interim efficacy. Based on the regression models, the predicted treatment effects of LLLT, as indicated by the mean reduction in VAS scores, are equal to 37.47–0.213 × (energy density) for immediate post-treatment observations and 44.45–0.166 × (energy density) for evaluations 1–3 months after treatment. Each unit of increase in ‘energy density’ contributes to a 0.213 or 0.166 decrease in the VAS score of the LLLT-based alleviation of DH in terms of the immediate or interim efficacy, respectively (Table 4).

**Table 4:** Regression models based on random-effect model for the effects of LLLT on DH alleviation. A) immediate efficacy; B) interim efficacy

A Immediate efficacy								
Covariates	Complete model				Final model			
	*B*	SE	95% CI	Sig	*B*	SE	*β*	Sig
Energy density	− 0.375	0.126	− 0.656, − 0.093	0.014	− 0.213	0.082	− 0.389, − 0.038	0.021
Risk of bias_moderate	4.211	8.238	− 14.144, 22. 566	0.620				
Risk of bias_high	8.066	8.134	− 10.058, 26.190	0.345				
Wave mode	− 6.960	9.475	− 28.072, 14.152	0.479				
Total dosage	0.263	0.180	0.137, 0.663	0.174				
Wavelength	0.029	0.039	− 0.059, 0.117	0.477				
(Constant)	8.855	29.262	− 56.345, 74.055	0.768	37.477	3.619	29.763, 45.191	0.000
Num. of observations	17				17			
*τ*^2^	108.7				99.38			
*I*^2^_*res*_	80.84%				83.89%			
Adjusted *R*^2^	28.58%				34.71%			
B Interim efficacy								
Covariates	Complete model				Final model			
	*B*	SE	95% CI	Sig	*B*	SE	*β*	Sig
Energy density	− 0.194	0.063	− 0.357, − 0.031	0.028	− 0.166	0.058	− 0.295, − 0.037	0.017
Risk of bias_moderate	− 3.902	4.680	− 15.933, 8.129	0.442				
Risk of bias_high	4.509	2.606	− 2.189, 11.208	0.144				
Wave mode	− 6.829	4.801	− 19.170, 5.512	0.214				
Total dosage	0.125	0.094	− 0.117, 0.366	0.242				
Wavelength	0.007	0.021	− 0.046, 0.060	0.757				
(Constant)	38.255	15.378	− 1.275, 77.786	0.055	44.452	2.22	39.501, 49.404	0.000
Num. of observations	12				12			
*τ*^2^	0				18.28			
*I*^2^_*res*_	0.00%				49.11%			
Adjusted *R*^2^	100%				60.11%			

#### DH-alleviating efficacy of LLLT compared to other in-office desensitisation strategies

**Fig. 5:**
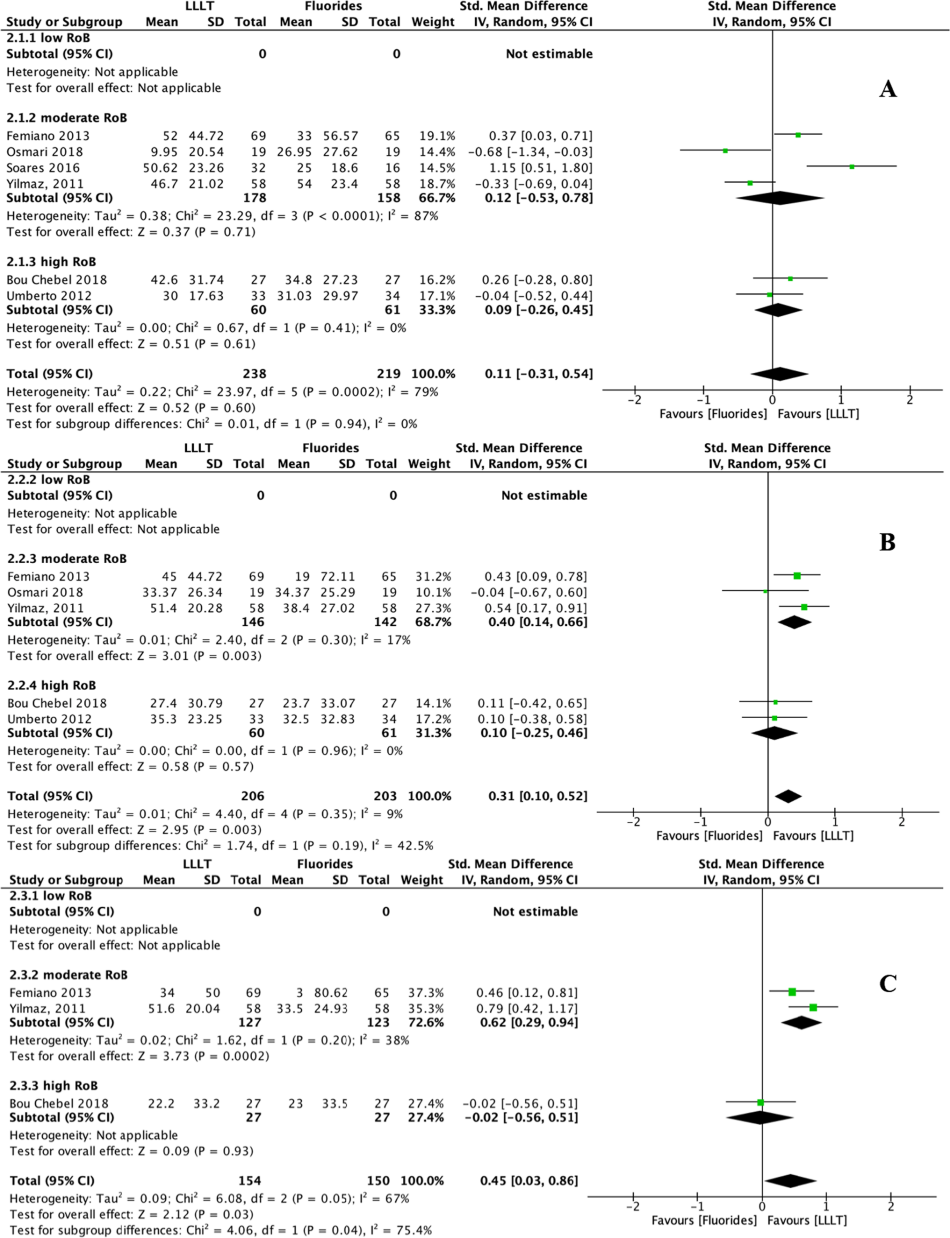
Forest plots indicating treatment efficacy of LLLT on DH alleviation compared to fluorides: **A** immediate efficacy; **B** interim efficacy; **C** persistent efficacy

**Fig. 6:**
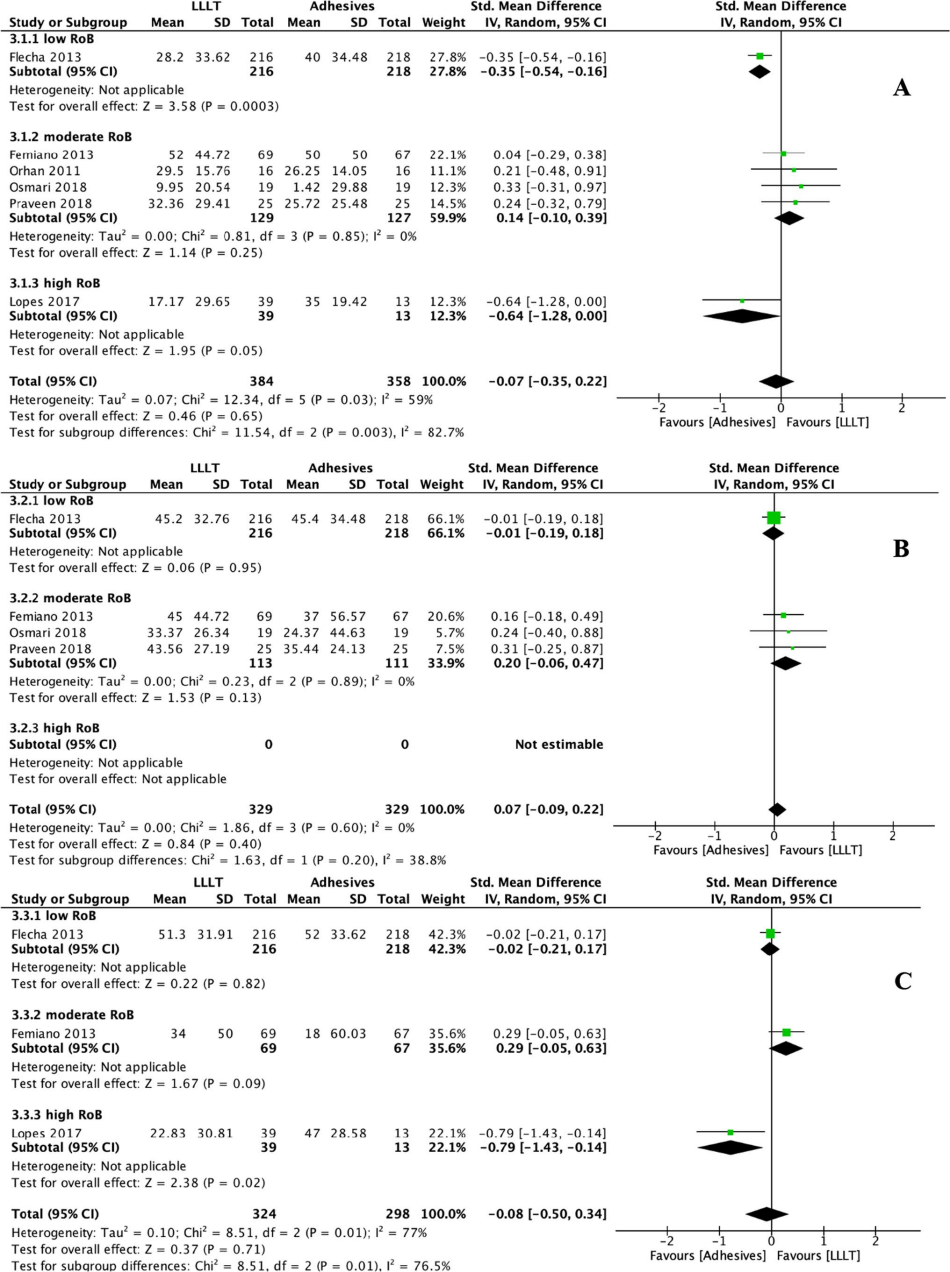
Forest plots indicating treatment efficacy of LLLT on DH alleviation compared to adhesives: **A** immediate efficacy; **B** interim efficacy; **C** persistent efficacy

**Fig. 7:**
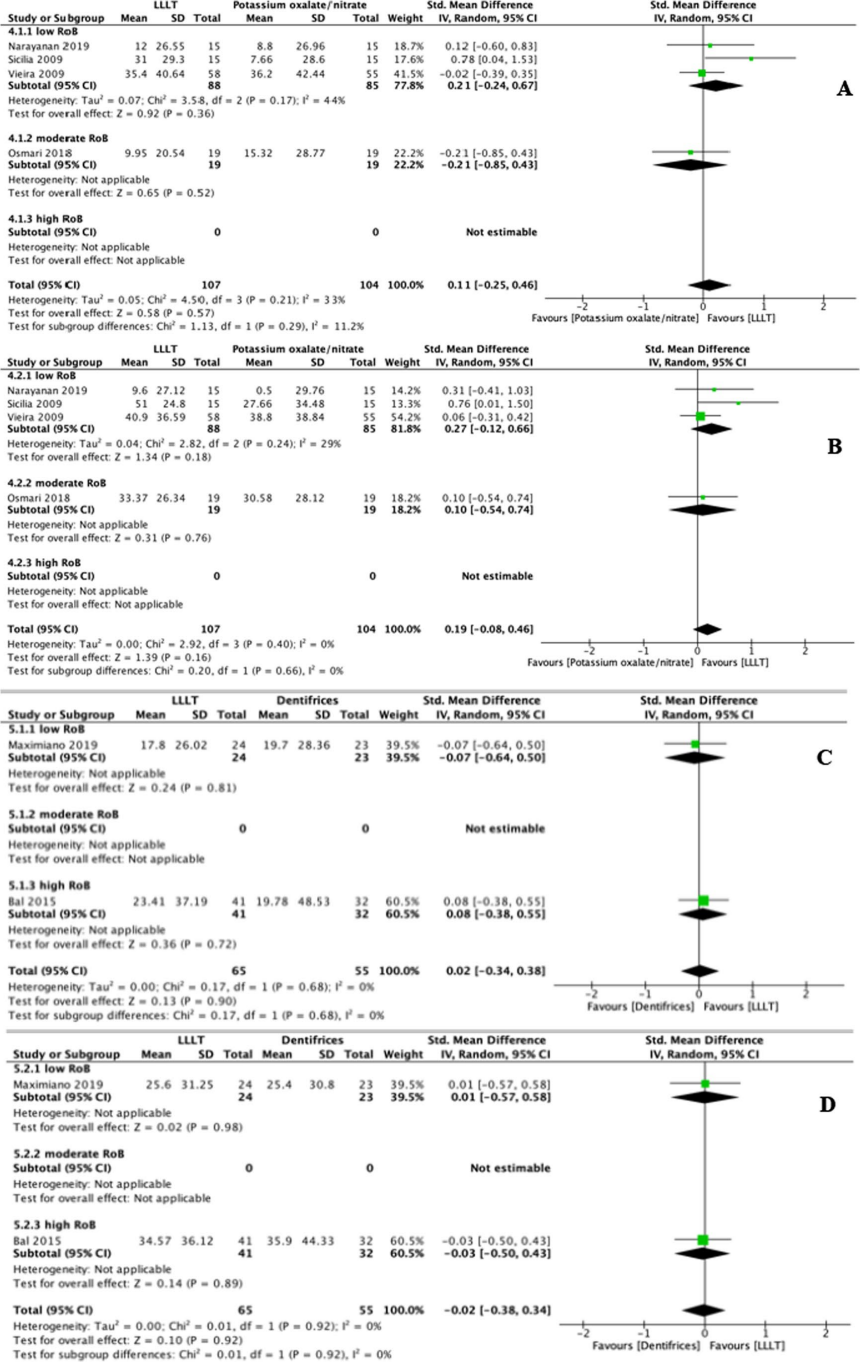
Forest plots indicating treatment efficacy of LLLT on DH alleviation compared to potassium compounds: **A** immediate efficacy; **B** interim efficacy; and to dentifrices for **C** immediate efficacy; **D** interim efficacy

In addition to placebo, the VAS changes in response to air blasts were also compared between LLLT and other in-office desensitisation agents, namely fluorides, adhesives, potassium compounds, and dentifrices. To make it align with the other groups for consistency of statistical analysis method, we still performed a subgroup analysis for these outcomes. Compared to fluorides, LLLT had no DH-alleviating effect in terms of immediate efficacy (SMD: 0.11, 95% CI: − 0.31 to 0.54, *p* = 0.60) but yielded slightly higher interim (*p* = 0.003) and persistent efficacies (*p* = 0.03) (Fig. 5). Interestingly, we noticed that when comparing immediate and interim efficacies between LLLT and fluorides, the heterogeneity in the moderate RoB subgroup (*I*^2^: 87% and 17% for immediate and interim efficacy, respectively) was even more considerable than the total heterogeneity (*I*^2^: 79% and 9% for immediate and interim efficacy, respectively). This result could relate to the minimal number of studies (*n* = 6) addressing fluorides comparator and no study with low RoBs. Compared to adhesives, LLLT had no DH-alleviating effect at any stage (*p* > 0.05) (Fig. 6). Similar results were obtained for comparisons with potassium compounds and dentifrices; for these, the SMDs of LLLT range from − 0.02 to 0.19 for immediate and interim DH-alleviating efficacy, with no statistically significant difference (*p* > 0.05), and no persistent efficacy data could be synthesised (Fig. 7). However, these results must be interpreted with caution, given the considerable heterogeneity within subgroups and the inclusion of few RCTs with a low risk of bias and few studies that addressed persistent efficacy.

## Discussion

One novelty of this systematic review and meta-analysis is that we conducted stage-based analysis on LLLT’s desensitising effects. Although the biomolecular and cellular PBM activities have not been entirely determined, there were three perspectives referring to different stages how LLLT alleviates DH. First, LLLT may immediately change patients’ self-perception by modulating neuronal physiology in terms of varying the axonal flow, cytoskeletal organisation, and adenosine triphosphate production in sensory nerves [68–70]. Second, the effect of LLLT on inflammation may play a role in the interim alleviation of DH since studies suggested there is a potential relationship between DH and micro-inflammation within dentine-pulp complexes [71–74]. The third theory is more explicable for persistent DH relief, as light irradiation may help increase blood vasculature in pulp tissues and stimulate the viability of odontoblasts; they both contribute to the deposition of secondary dentine and reduction of dentin permeation [75–78]. Based on the above three theories, we investigated the DH-alleviating efficacy of LLLT treatment in a stage-based manner and separately extracted data for immediate, interim, and persistent outcomes. Another intention of using the stage-based data synthesis is to reduce the clinical heterogeneity of included studies and avoid correlation-associated overestimations. Marto et al. [25] adopted the same strategy; unfortunately, they included all laser types as one desensitising approach and did not elaborate on the effects of LLLT. Another two systematic reviews did examine different types of laser therapies [19, 79]; yet they only retrieved data of the earliest and latest time points without consideration of the association between clinical performance and biological activities underneath.

Another novelty of our systematic review is that we performed a methodological subgroup analysis to investigate the causes and type of heterogeneity [80]. Specifically, the analysis of LLLT’s efficacies was based on the quality assessment of included studies. Intuitively, studies with low RoBs provide the highest quality and should play the dominant role in generalisation. However, prerequisites should be sufficient high-quality evidence and acceptable heterogeneity to avoid loss of power or dilution of efficacy estimates [80]. Among the included studies for immediate efficacy, only three RCTs had low RoBs but presented high heterogeneity (*I*^2^: 49%), while seven studies had moderate or high RoBs with relatively mild heterogeneity (*I*^2^: 36% and 0%, respectively). Therefore, we also included studies with moderate and high RoBs for meta-analysis to obtain a more general overview of the results, and demonstrated outcomes by their quality.

In addition, this systematic review further conducted a meta-regression to examine the causes of heterogeneity and explore confounding factors [81]. Out of five factors that potentially relate to VAS changes, we only found ‘energy density’ was significantly associated with immediate and interim efficacies. Energy density (J/cm^2^), also called ‘fluency’, is a crucial parameter in LLLT and represents the energy absorbed by tissues per unit area [82]. In vitro and in vivo studies have reported a close relationship between the energy density of irradiation and the biphasic responses of a patient in terms of the stimulation or inhibition of biological activities [83–85]; an optimal energy density generates the maximum desired PBM [13]. Notably, our meta-regression results support their findings: LLLT has a higher immediate and interim DH-alleviating efficacy under low energy density (2–10 J/cm^2^) in comparison with those under higher energy density (> 40 J/cm^2^). However, a lack of data prevented us from determining the optimal DH-alleviating energy density, as many reports lacked detailed information on LLLT settings [42–44, 53, 54, 57]. Also, the negative correlations of regression models should be interpreted with great caution, as substantial residual variances of 83.39% and 49.11% were observed for immediate and interim efficacy, respectively.

Overall, this systematic review bridges a critical research gap by analysing current clinical evidence in the DH-alleviating efficacy of LLLT. Despite striving for a pertinent data synthesis plan and meta-analysis method, the following limitations exist. First, the number of well-conducted RCTs with high quality was quite insufficient. There were only three studies with low RoBs available for comparison between LLLT and placebo, which presents relative high heterogeneity, i.e. 49% and 64% for immediate and interim efficacy, respectively. In addition, the absence of studies with low RoBs on the efficacy difference between LLLT and fluorides indicates that more studies are required to warrant convincing evidence in the future. Second, there is a great inconsistency in the age range for recruited subjects and intervention/assessment methods for LLLT and its comparators. Third, quantitative analysis on DH was only conducted on the air blast–stimulated response due to insufficient and inconsistent data for other clinical outcomes. Finally, and there is a shortage of studies that cover long-term follow-ups. These may bring substantial bias in evaluating persistent efficacy when the technical settings of LLLT were divergent [6]. Therefore, we advocate more well-conducted RCTs with low RoBs, consistent settings, comprehensive assessments, and long follow-up periods in the future to generate high-quality evidence regarding the DH-alleviating effects of LLLT.

## Conclusion

This systematic review analysed clinical evidence regarding the DH-alleviating efficacy of LLLT. The immediate, interim, and persistent efficacy results show that, compared to placebo, LLLT generally alleviated DH in the included studies. Energy density appears to be a critical factor for the successful treatment of DH with LLLT, as higher immediate and interim efficacy was achieved under low-energy–density conditions. The evidence does not suggest that the DH-alleviating effects of LLLT are superior to those of other in-office desensitisation strategies, except fluorides in terms of interim and persistent efficacy. Future RCTs with low RoBs, consistent settings, comprehensive assessments, and long follow-up periods are highly recommended.

## Supplementary Information

Below is the link to the electronic supplementary material.

Supplementary file1 (DOCX 17 KB)
