# Supplementary material for: Effects of low-level light therapy on dentin hypersensitivity: a systematic review and meta-analysis
Source: Clin Oral Investig. 2021 Oct 13;25(12):6571–95. doi: 10.1007/s00784-021-04183-1 (PMC8602177; doi:10.1007/s00784-021-04183-1)
Supplement: Supplementary file 1 — Supplementary file1 (DOCX 17 KB) [file 784_2021_4183_MOESM1_ESM.docx]

***Appendix1***

| Medline  65  EMBASE  199 | 1. Tooth/ 2. exp Dental Pulp Necrosis/ or exp Dental Pulp/ or exp Dental Pulp Test/ 3. exp Dentin/ 4. 1 or 2 or 3 5. (sensitiv* or hypersens* or hyposens* or desens* DH).mp. 6. (feel* or percept* or irritat* or pain* or numb* or discomfort* or complain*).mp. 7. (visual assessment score or VAS or vitality test*).mp. 8. (cold test* or hot test* or electric test*).mp. or exp Dental Pulp Test/ 9. 5 or 6 or 7 or 8 10. (Low-level laser or low-intensity laser or low-power laser or diode laser or biomodulat* or photobiomodulat* or PBM or soft laser or cold laser or phototherap* or light therap* or therapeutic laser).mp. 11. 4 and 9 and 10 |
| --- | --- |
| CENTRAL  729 | 1. tooth or teeth or dentin or pulp 21981 2. sensitiv* or hypersensit* or hyposensit* or DH or desensit* or feel* or percept* or irritat or pain* or numb* or discomfort* or complain* or visual assessment score (VAS) or vital* or cold test or hot test or electric pulp test 1617981 3. #2 not (cari* or cavit* or decay*) 1593464 4. Low-level laser or low-intensity laser or low-power laser or diode laser or biomodulat* or photobiomodulat* or PBM or soft laser or cold laser or phototherapy* or light therap* or therapeutic laser 22310 5. #1 and #3 and #4 in Trials 729 |
| PubMed  84 | (((tooth OR dentin OR pulp) AND (sensitiv* OR hypersensit* OR hyposensit* OR DH OR desensit* OR feel* OR percept* OR irritant OR pain* OR numb* OR discomfort* OR complain* OR visual assessment score (VAS) OR vital* OR cold test OR hot test OR electric pulp test)) NOT (cari* OR cavit* OR decay*)) AND (low-level laser OR low-intensity laser OR low-power laser OR diode laser OR biomodulat* OR photobiomodulat* OR PBM OR soft laser OR cold laser OR phototherapy* OR light therap* OR therapeutic laser) |
| Scopus  43 | ( ( tooth OR dentin OR pulp ) AND ( sensitiv* OR hypersensit* OR hyposensit* OR dh OR desensit* OR feel* OR percept* OR irritat OR pain* OR numb* OR discomfort* OR complain* OR visual AND assessment AND score OR vas OR vital* OR cold AND test OR hot AND test OR electric AND pulp AND test ) AND ( low-level AND laser OR low-intensity AND laser OR low-power AND laser OR diode AND laser OR biomodulat* OR photobiomodulat* OR pbm OR soft AND laser OR cold AND laser OR phototherapy* OR light AND therap* OR therapeutic AND laser ) ) AND NOT ( cari* OR cavit* OR decay* ) |
| ProQuest  432 | (Tooth or dentin or pulp) AND (sensitiv* or hypersensit* or hyposensit* or DH or desensit* or feel* or percept* or irritat or pain* or numb* or discomfort* or complain* or visual assessment score or VAS or vital* or cold test or hot test or electric pulp test) AND (Low-level laser or low-intensity laser or low-power laser or diode laser or biomodulat* or photobiomodulat* or PBM or soft laser or cold laser or phototherapy* or light therap* or therapeutic laser) NOT (cari* OR cavit* OR decay*) |
| Total | 1552-124 duplicates = 1381 |
